# Active elements, effects, and working mechanisms of creative arts therapies in forensic psychiatric care: a realist review

**DOI:** 10.3389/fpsyt.2025.1745054

**Published:** 2026-01-23

**Authors:** Susan van Hooren, Annemarie Abbing, Wim Waterink

**Affiliations:** 1 Academy of Creative Arts Therapies and Psychomotor Therapy, Zuyd University of Applied Sciences, Heerlen, Netherlands; 2 Faculty of Psychology, Open University, Heerlen, Netherlands; 3 KenVaK, Research Centre for the Creative Arts Therapies and Psychomotor Therapy, Heerlen, Netherlands; 4 Department of Arts Therapies, Faculty of Health, University of Applied Sciences, Leiden, Netherlands

**Keywords:** art therapy, creative arts therapies, dance-movement therapy, drama therapy, forensic, music therapy, realist review

## Abstract

**Introduction:**

Mental disorders are highly prevalent among forensic detainees, complicating treatment and increasing recidivism risk. Due to limited insight and communication difficulties, experiential approaches, such as creative arts therapies are needed. Creative arts therapies - including art therapy, music therapy, drama therapy, and dance/movement therapy - use psychotherapeutic engagement with art modalities to achieve therapeutic goals. As empirical studies demonstrate their effectiveness, questions shift from whether these interventions work to how and why they work.

**Methods:**

In this review, we applied principles of realist review approach to analyze how and why CATs work by identifying active elements and working mechanisms, aiming to clarify causal pathways and inform targeted clinical application.

**Results:**

Twenty-seven studies were included with 1437 participants in total. Based on realist review standards, four studies were excluded for relevance and six for rigor, leaving 17 studies included in the review. The analyses resulted in twelve models identifying active elements of arts therapies in forensic care, and explaining how these contribute to observed outcomes. Overall, a hierarchical structure can be observed, in which the identified active elements range from those grounded in fundamental physical and bodily experiences, to those facilitating emotional expression and recognition, progressing further to elements that engage higher-order cognitive processes -such as attention regulation and executive or inhibitory functioning- and culminating in elements that foster social interaction and prosocial behavior.

**Discussion:**

These models offer a conceptual framework for understanding creative arts therapies in forensic contexts and guide further empirical research and refinement of clinical practice.

## 1 Introduction

Mental disorders are highly prevalent among detainees in forensic care and are associated with an increased risk of recidivism. Many individuals in forensic and correctional settings suffer from comorbid conditions, including personality disorders, psychosis, intellectual disabilities, aggression, and trauma histories. These factors complicate treatment and rehabilitation and elevate societal risk when left unaddressed (1). Treatments in forensic and correctional settings are predominantly verbal, yet this population often exhibits limited insight into own behavioral patterns, problems in self-reflection, and communication difficulties, necessitating other approaches. Experiential approaches, such as creative arts therapies, can be considered as these offer action-based experiences in the “here and now” guided by a therapist, which can offer offenders an experiential insight into their own behavior and promote motivation for treatment.

Creative arts therapies—including art therapy, music therapy, drama therapy, and dance/movement therapy—use psychotherapeutic engagement with art modalities to achieve therapeutic goals (2). These interventions facilitate the externalization of behavioral patterns, capacities, and limitations, thereby informing both diagnostic processes and individualized treatment planning. Due to their experiential and action-oriented approach, creative arts therapies enable therapeutic work on key forensic objectives such as emotional regulation, impulse and aggression control, development of empathy, and enhancement of interpersonal functioning. This approach can promote the expression of feelings and emotions, such as remorse or a desire to belong (e.g. 3). It can validate personal experiences and emotions, including grief, and support meaning-making and a sense of control (4). Additionally, it encourages accepting responsibility, acknowledging past wrongdoings, and feeling seen and appreciated (3, 5) Their non-verbal methods allows engagement with affective content that may be too threatening through verbal means, which is especially valuable for patients with low intellectual functioning or limited verbal communication. The importance of these therapies in forensic and correctional settings is increasingly acknowledged by clinicians, service users with psychiatric disorders, and policymakers (6, 7).

A recent review and meta-analysis on 18 effect studies (RCT, CCT, and pre-post designs) showed that creative arts therapies reduce psychiatric symptoms and improve social and psychological functioning confirming their effectiveness in both forensic as well as correctional settings (8). As creative arts therapies are increasingly acknowledged in clinical and forensic contexts, and empirical studies demonstrate their effectiveness, questions shift from whether these interventions work to how and why they work. A previous narrative synthesis proposed a number of working mechanisms including emotion regulation, stress regulation, impulse regulation, cognitive regulation, social regulation, behavior regulation, and self-management (8). However, this synthesis was based on a narrative analysis, a more thorough and systematic analyses is warranted to deepen understanding.

To address explanatory questions on how and why, a realist review was introduced (9) and embraced across domains, such as public health domain, clinical psychology, nursing, and medicine (e.g. 10, 11). Realist review is characterized by its theory led approach and its focus on generative causation: the idea that observable outcomes are produced by underlying, often unobservable, working mechanisms operating within specific contextual conditions. It is particularly suited for unpacking the complexity of interventions composed of multiple interacting components and contextual variables. Creative arts therapies exemplify such complex interventions (12), referring to multi-faceted, targeting a range of behaviors, involving numerous interacting elements that may often require tailoring or adaptation, and which is subject to the influences of those delivering (skills and expertise) and receiving the intervention with differing results as a consequence. Grounded in the principles of realist review methodology, the realist review aims to achieve ontological depth by theorizing beyond empirical outcomes to uncover the working mechanisms that generate observed therapeutic effects. In realist review, context, working mechanism, and outcome configurations are developed that help to explain of how and why an intervention works. These configurations are in essence theories on the relation between working mechanisms and context that bring about a particular outcome (13).

Arts therapies interventions are considered as complex interventions, because they involve multiple interacting components, address diverse behaviors and goals, apply to different patient groups, yield various outcomes, and require flexibility or tailoring (12, 14). The interventions are multifaceted with specific therapeutic actions and procedures carried out by the arts therapist. These therapist-driven actions constitute an active element of the intervention, i.e. a visible component, aspect, or characteristic of the intervention that is deliberately applied by the therapist in a specific manner and contributes to a particular therapeutic effect. Following Abbing et al. (15, 16), these active elements can be categorized into specific active elements, i.e. those uniquely associated with particular arts therapy disciplines and non-specific active elements, which are not exclusive to a single discipline, e.g. therapeutic attitude. An element is considered active when it contributes to a measurable effect, meaning a significant change resulting from an intervention, as measured by a relevant outcome measure (see also, 15, 16). The causal relationship between an active element of an intervention and the effect can be explained by a working mechanism. A working mechanism refers to internal processes through which an intervention exerts its effect. In general, working mechanisms are regulatory brain networks, physiological and/or psychological processes, that underlie the observed effects.

In this review, we applied principles of realist review approach to analyze creative arts therapies that have demonstrated effectiveness in forensic populations. The identified characteristics of these interventions serve as the foundation for delineating active elements. By systematically linking these elements to observed outcomes and the underlying working mechanisms believed to generate them, we constructed theoretical configurations that elucidate causal pathways within creative arts therapy interventions. These configurations enable a shift from simply asking whether an intervention is effective, to understanding why, how, and for whom it is effective. As theoretical constructs, they provide a foundation for more targeted empirical investigations in the future and offer a refined lens through which to study and develop creative arts therapies in forensic and other complex care contexts.

## 2 Materials and methods

### 2.1 Design

**Figure 1:**
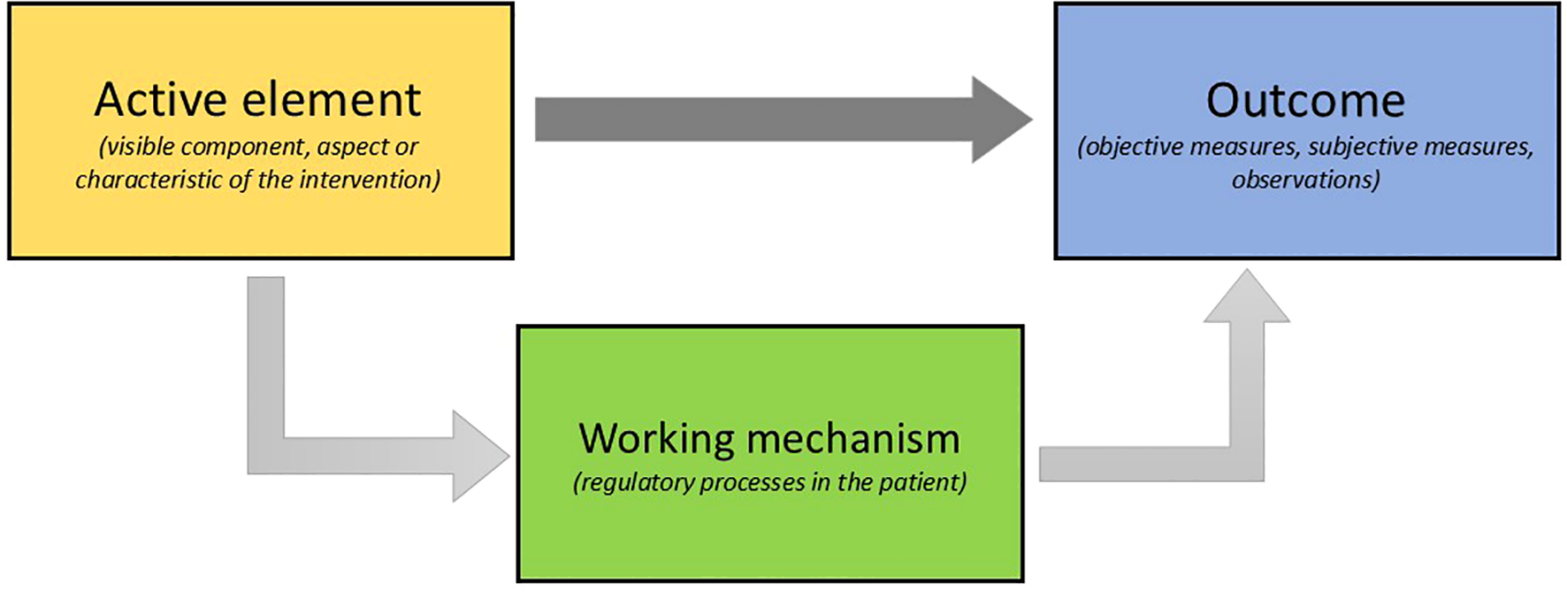
Adapted framework of the Realist Review: from active element to working mechanism to outcome of arts therapies.

The literature review was conducted according to the principles of the Realist Review (RR) (9, 17). A RR seeks to identify what works, for whom, in what circumstances, and why, by developing and refining theories expressed as context-mechanism-outcome (CMO) configurations. In line with realist review methodology, we sought to explain how and why arts therapies may contribute to rehabilitation in forensic care. Traditionally, realist reviews build context–mechanism–outcome (CMO) configurations (9). In the present review, we adapted this structure to match our specific aim: identifying active elements of arts therapies in forensic care, and explaining how these contribute to observed outcomes. Accordingly, our analysis followed the sequence: active element → working mechanism → outcome (**Figure 1**), adjusting the CMO framework.

The method was characterized by: 1) building on an existing framework, 2) systematically searching for and evaluating evidence (i.e. a review), 3) an iterative process involving integrating perspectives from multiple disciplines (creative arts therapies, neuropsychology, theoretical and forensic psychology), and 4) adjusting and refining the framework when new insights emerge.

In this review we built on the earlier review by Abbing et al. (15). The protocol for this approach was registered in PROSPERO under number CRD42020217884 (18). In the RR, our analysis was based on (a) the interventions described in the included studies, (b) the assumptions authors articulated about how the interventions work, (c) the empirically observed effects, and (d) models drawn from forensic care, neuroscience, and psychology. From each included study, active elements, working mechanisms, and effects were extracted and subsequently connected to one another, see **Figure 1**.

### 2.2 Search and selection of the studies

The search strategy combined terms related to each creative arts therapy discipline with terms related to forensic setting and forensic psychiatric populations (see Appendix X for the search terms). The following databases were searched: CINAHL, Embase, Medline, PsycINFO, and Web of Science (WOS). The initial search was conducted in June 2020, with an update in January 2023 and Octobre 2025. We followed PRISMA protocol for reporting the search and selection process (19).

The selection of articles occurred in two steps: 1) based on title and abstract, and if deemed relevant, 2) based on the full text. The selection of studies was carried out independently by two researchers. In both steps, each study was assessed independently by two reviewers. In case of discrepancies, consensus was sought. If consensus could not be reached, a third researcher was consulted to provide an independent judgment. A final decision was then made based on the collected arguments. The same inclusion and exclusion criteria as used in the previous review were applied in this selection process (15):

- language (English, Dutch, and German),
- year of publication (>1980),
- peer-reviewed, design (Randomized controlled trial, clinical controlled trial, case control studies, cohort studies and multiple case studies),
- comparator (no treatment/waiting list, inactive control, active control (e.g. CBT), no control),
- population (adults aged 18 till 65 years with mental health problems in a correctional setting or a forensic institution, with or without mental health services),
- intervention (art therapy, music therapy, drama therapy and dance movement therapy, provided by an arts therapist).

We excluded interventions without a trained arts therapist. A trained arts therapist has received a bachelor of master educational program in the field of one of the arts therapies. When therapy was only part of a broader program primarily delivered by other health care professionals, it was included in the review only if the creative arts therapy component was described in the study and delivered by a trained arts therapist. The type of outcome measures were restricted to quantitative measures of psychosocial outcomes.

In this review, we only considered statistically significant (p < 0.05) outcomes reported in the included studies. The rationale for this decision is that the identification of an active element, as a trigger of an underlying working mechanism, is only relevant when there is an observable effect. Non-significant findings may be valuable in understanding study limitations or contextual influences, but they cannot substantiate a link between a therapeutic intervention, the hypothesized underlying working mechanism and the resulting outcome. By focusing on significant results, we ensured that the extracted frameworks, active element–working mechanism–outcome, were grounded in demonstrable effects.

Following realist review standards (9, 17), each study was also judged on 1) Relevance: the extent to which the study could contribute to developing, supporting, or refining CMO configurations and 2) Rigor: whether the study’s methods were sufficiently credible to warrant drawing inferences. Studies with lower methodological rigor, but high relevance were retained as potential sources for theory building, whereas those with stronger rigor were weighted more heavily when linking active elements- working mechanisms- outcomes.

### 2.3 Data-extraction

We extracted the following data from the included studies: Creative arts therapy discipline, specific characteristics of the intervention, duration of the intervention, frequency of the sessions, if mentioned, active elements and working mechanisms (described or implied), outcome measure (primary and secondary), results (difference between groups as well as within groups), effects (descriptive). Data extraction was performed by three researchers (AA, SH, WW), who separately extracted the data from included publications using a standard extraction sheet (**Supplementary Material 2**). Random checks were performed by one of the researchers to ensure consistency.

### 2.4 Analyses

In accordance with the principles of Realist Review (RR), the leading extracted components were systematically linked to one another. These components included: 1) active elements, 2) working mechanisms, and 3) effects. With regard to the active elements, the analysis was based on the extracted specific characteristics of the intervention. For the working mechanisms, the typology of arts therapy mechanisms described by Van Hooren et al. (20) was used as a starting point. This typology organizes potential working mechanisms drawn from the field of arts therapy and related scientific domains, such as neuroscience, musicology, criminology, and clinical, neurodevelopmental, and forensic psychology and psychiatry. When the results of the literature review indicated the need, this typology was expanded and/or adapted for forensic care. The effects were clustered in accordance with the previously applied clustering method (15). This clustering is based on the Risk-Needs-Responsivity model (21), the Good Lives Model (22), and the HKT-R (23). It consists of three clusters of risk factors (i.e., criminal and antisocial behavior, addiction, and psychiatric symptoms) and two clusters of protective factors (i.e., social and relational functioning, and psychological functioning).

First, for each study, the active elements, effects, and working mechanisms were linked to one another. In a second step, integration took place. During this integration, the active elements for each intervention were clustered based on content. For each cluster, a relationship was established with demonstrated effects that aligned with expectations, as well as with potential working mechanisms.

## 3 Results

### 3.1 Study selection and characteristics

**Figure 2:**
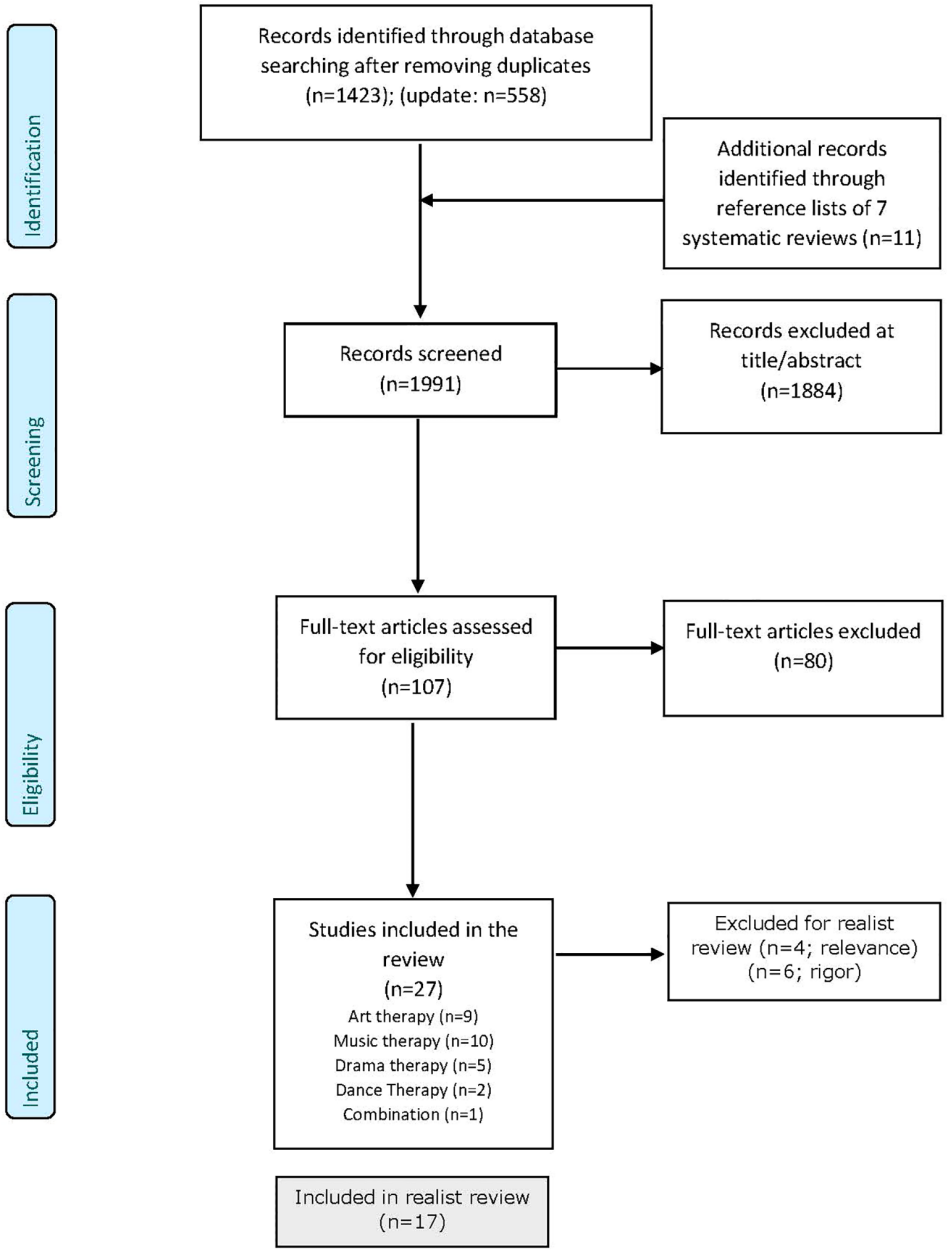
PRISMA flow diagram.

The first search in 2023 resulted in 1423 unique citations and the update of the search in 2023 and 2025 yielded 558 additional citations. These searches yielded seven systematic reviews. Screening of the titles of the reference lists of these reviews resulted in 11 extra citations that were eligible for further screening. Based on screening all titles and abstracts, using the in- and exclusion criteria, 1874 records were excluded. In a second phase, we screened the full texts of the remaining 107 records. This resulted in 27 included studies, see **Figure 2**.

The 27 studies included 1437 participants with sample sizes ranging from 1 to 200. There were nine studies on art therapy, ten on music therapy, five on drama therapy, two on dance-movement therapy, and one study examined an intervention with different creative arts therapies. Seven studies had a RCT design, six a CCT design, thirteen a pre-post design, and one had a single case design. Ten studies concerned a forensic/correctional setting and 17 studies took place in a forensic care setting. Based on the realist review standards, four studies were additionally excluded based on relevance (i.e. only small part of the studies intervention consisted of creative arts therapies elements; no effect study with pre- and post-treatment measures; previous published data) and six studies based on rigor (i.e. statistical significance was not p < 0.05, p-value not reported), resulting in 17 studies included in the realist review. For an overview of these studies and the characteristics of the interventions, active elements and its clustering, assumed working mechanisms, and effects found in these studies, see **Table 1**.

**Table 1:** Active elements, mechanisms, and effects of analyzed studies.

Author - Year	Type	Intervention characteristics	Active element	Clustering of elements	Mechanism	Ouctome
Chen, et al. (24)	MT	music improvisation in a group; writing a song	(creative) expression	creative expression	emotion regulation	anxiety/depression
		music improvisation in a group	attuning to another person	attunement	regulation of social processes	self-esteem
		music listening; music improvisation in a group; writing a song	working together in a group	Working together in a group	regulation of social processes	self-esteem
Gussak (25)	AT	drawing; claying; poetry	(creative) expression	creative expression	emotion regulation	depression
		drawing; claying; poetry; group activities; working together	reflection	reflection	regulation of cognitive processes	control
		drawing; claying; poetry; group activities; working together	positive dynamic social interactions	Working together in a group	regulation of social processes	pro-social behavior
Gussak (26)	AT	drawing; claying; poetry; creating an outside-inside box	(creative) expression (non-verbal); symbolization	creative expression	emotion regulation	depression
		drawing; claying; poetry; creating an outside-inside box	symbolization	symbolization	emotion regulation	depression
		drawing; claying; poetry; creating an outside-inside box; group activities; working together	reflection	reflection	regulation of cognitive processes	motivation
		drawing; claying; poetry; creating an outside-inside box; group activities; working together	positive dynamic social interactions (including humour)	Working together in a group	regulation of social processes	pro-social behavior
Gussak (27)	AT	drawing in a group, as part of that group; working together; drawing an ideal world in a group	positive dynamic social interactions	working together	regulation of social processes	pro-social behavior
Hakvoort, et al. (28)	MT	Psychoeducation	psychoeducation	psychoeducation	learning	coping
Jeon, et al. (29)	MT	making the sounds using beating drum, in particular repetitive lower frequency-rhythms	changes in tempo	deactivation	arousal regulation	psychotic symptoms
Keulen-de Vos et al. (30)	DT	expressing basic emotions; role playing; role reversal	playing emotions	exploration	affect regulation	emotional sensitivity
Kellet et al. (31)	MT	music improvisation in group	working together in a group	Working together in a group	regulation of social processes	Interpersonal functioning
Koch et al. (32)	DMT	stick fighting	stops of thoughts and actions	inhibition	impulse regulation	Agression/anger
MacFarlane, et al. (33)	MT	Abdominal breathing techniques, rhythmical entrainment	relaxation	deactivation	arousal regulation	ptsd symptoms
		bilateral movement patterns in music with body percussion, and musical attention control training (i.e. training sustained attention by keeping to a rhythmic pattern and training selective attention by providing a competing musical stimulus, while the patient must maintain his rhythmic pattern)	musical excercises for attention	attention (divided / selective)	attention regulation	attention
		Psycho-education on common reactions to traumatic events and the brain and stress	psychoeducation	psychoeducation	learning	ptsd symptoms
Qui et al. (34)	AT	personal drawing or painting; therapist actively connects drawing/painting with thoughts/memories, emotions/feelings, and circumstances of client	(creative) expression	creative expression	emotion regulation	anxiety, depression, anger, and negative psychiatric symptoms
Stallone (35)	DT	role playing; role reversal	creative expression	creative expression	emotion regulation	incidents
		role playing; role reversal; sharing emotions; interacting with others	self-reflection	reflection	regulation of cognitive processes	incidents
Testoni et al. (36)	DT	role playing; role reversal	Expression (story telling) of emotions, feelings	creative expression	emotion regulation	addiction self-management
		role playing; role reversal; sharing emotions	cognitive restructurering	cognitive restructuring	Regulation of cognitive processes	daily functioning
		sharing emotions; interacting with others	observing others in a group	attunement	regulation of social processes	social regulation
Thaut (37)	MT	music listening; relaxation	relaxation	deactivation	arousal regulation	relaxation
		muscial expression; music improvisation	improvisation, strenghtening sense of agency	exploration	affect regulation	mood
		music listening; evoking memories; muscial expression; music improvisation	working together	Working together in a group	regulation of social processes	insight
Tsai (2024)	DMT	Breathing and regulation (orientation; energy modulation in varied movement activities; introduction of process model of emotion regulation; calming and breathing exercises), situation selection, and practicing appreciation	body oriented activity	body oriented activity	Allostatic regulation of the body	body appreciation
Yu, et al. (38)	AT	drawing; therapist actitively connects drawing/painting with thoughts/memories, emotions/feelings, and circumstances of client	drawing (house (projection of the family)-tree (environment)-person self-identification));	symbolization	emotion regulation	anxiety
Zeuch and Hillecke (39)	MT	monochordplay; singing	deactivation	deactivation	arousal regulation	emotional balance

AT, art therapy; DMT, dance-movement therapy; DT, dramatherapy; MT, music therapy.

### 3.2 Models

The analyses resulted in twelve clusters of active elements. For each cluster, there was a relation with examined outcome and proposed working mechanism. This yielded the following models between active element-working mechanism-effect:

1. Deactivation – arousal regulation – relaxation.

Several of the interventions described in the included studies contained deactivation as an active element. More specifically, these interventions contained relaxation exercises, such as breathing exercises, listening to relaxing music, and making sounds using beating drum with repetitive lower frequency rhythms (33, 37; 29, 39). These studies found that these interventions led to more relaxation, but also to more emotional balance and less symptoms of posttraumatic stress disorder and less overall psychiatric symptoms. The effect of deactivation can be explained by arousal regulation, meaning that the autonomic nervous system regulates arousal to meet both the individual’s internal state and environmental demands. This regulation of the nervous system’s activation state has been shown to influence symptomatology.

2. Body oriented activity – allostatic regulation of the body – body awareness.

In one study on dance movement therapy, subjects had to focus on bodily sensations (40). This body oriented activity may be the active element of increasing body acceptance and body appreciation found in this study. This effect may be explained by allostatic regulation of the body, where interoception plays an important role. Allostatic regulation refers to the process in response to external stimuli or anticipated changes in order to achieve and maintain homeostasis.

3. Exploration – affect regulation – mood/emotional vulnerability.

Exploration of own behavior, intentions, and roles was seen in several interventions. Concrete illustrations were seen in playing with basic emotions in drama therapy, enacting a scene of a situation in which a particular need was neglected or in which feeling of anger were present, role playing with rescripting to a more positive scene, and improvisation. Studies using these elements led to a better mood and more emotional vulnerability (30, 37). It is assumed that these effects can be explained by affect regulation, referring to internal processes on recognizing, comprehending, and modulating one’s own affective state.

4. Creative expression – emotion regulation – depressive complaints.

In several studied interventions, participants were able to express themselves through drawing, sculpting, and writing poetry (in art therapy; 26), through expressing basic emotions or role-playing (in psychodrama; 35; 36), and through making music or writing songs, such as replacing whole lyrics for a song or creating new melodies (in music therapy; 24). Creative expression is considered the active component in these approaches. This was associated with a reduction of negative emotions, such as anxiety and sadness, which was also reflected in less depressive symptoms, less disciplinary reports, and higher subjective wellbeing. These reductions of negative emotions after therapeutic creative expression is explained by improved emotion regulation, referring to internal processes on recognizing, understanding, and managing negative and also positive emotions.

5. Symbolization – emotion regulation – anxiety and depressive symptoms.

The next active element found in the interventions of the included studies was symbolization. Illustrations are drawing an ideal or desired world (26) and drawing specific themes, e.g. house, tree, person (38). The use of symbolization can engage internal processes through which emotions are recognized, understood, and regulated (i.e. emotion regulation). Studies showed that this led to a reduction in anxiety and depressive symptoms (26, 38).

6. Reflection – regulation of cognitive processes–control and motivation.

In several interventions, participants were challenged to reflect on their own behavior, intentions, and roles (25, 26, 35). Studies showed that reflection led to more control, more motivation, and from that to less disciplinary reports (25, 26, 35). It is assumed that these observed effects can be explained by regulation of cognitive processes refers to internal processes through which thoughts are consciously directed, controlled, or modified in order to more effectively manage various situations, emotions, tasks, or demands.

7. Attention training – attention regulation –selective and sustained attention.

Several interventions contained attention training components as active element. For instance, in music therapy, participants are instructed to maintain a specific rhythmic musical pattern in order to train sustained attention (33). Also, selective attention was trained by providing a competing musical stimulus, while the patient must maintain his rhythmic pattern (33). Studies showed that after these interventions selective- and sustained attention was improved in participants (33). This may be explained by improved regulation of attention. Attention regulation refers to internal processes that enable focusing on what is important (focused attention), ignoring distractions (selective attention), sustaining concentration (sustained attention), and dividing attention across multiple tasks (divided attention). Attention regulation is essential for daily functioning and for understanding how attention can support the development of attentional skills and strategies.

8. Inhibition – impulse regulation –anger.

Inhibition as an active element was seen in a dance movement therapy intervention using stick fighting, in which participants need to stop their thoughts and actions to establish interpersonal boundaries. This led to a reduction in anger among participants after the intervention (32). This observed effect may be explained by improved regulation of impulses. Impulse regulation refers to internal processes that control sudden or automatic reactions, particularly when such responses are misaligned with personal goals or social norms. Impulse regulation plays a critical role in social contexts, where impulsive behaviors—such as outbursts of anger—may lead to undesirable consequences. Effective impulse regulation helps prevent individuals from responding inappropriately.

9. Psycho-education – learning – coping.

Several interventions contained elements of psycho-education provided by creative arts therapists. In these elements more information was given on the different phases of anger (28). Another illustration was on providing more information about common reactions to traumatic events and about brain functioning and stress (33). Information was basic and attuned to the intellectual level of the participants (28, 33). By providing concrete knowledge using psycho-education, stress symptoms could be reduced through learning, or individuals could learn to cope more effectively with stress.

10. Cognitive restructuring – regulation of cognitive processes – daily functioning.

Cognitive restructuring was applied as an element in several interventions. In cognitive restructuring, irrational or maladaptive thoughts were identified, questioned, and replaced with or realistic or positive alternatives. This was applied by role playing in dramatherapy (36). A characteristic feature of role-play is the use of imagination, play, and exaggeration, which challenges alternative perspectives and thoughts about situations. Cognitive restructuring contributed to improvements in daily functioning, with the regulation of cognitive processes considered a key underlying mechanism.

11. Attunement – regulation of social processes – social functioning.

In several interventions, therapists focused on attunement to others. This was seen as an active element in improvisations in group sessions using role playing (36) or musical improvisations (24). After these interventions, results showed better social functioning, that may result in a higher self-esteem. These observed effects may be explained by regulation of social processes, which refers to internal mechanisms involved in understanding, guiding, and influencing interactions and relationships with others.

12. Working together in a group – regulation of social processes – pro-social behavior, empathy, sensitivity to others.

Working together in a group was seen as an active element in several interventions. This was seen in an art therapy intervention, in which a group created an art product together (25, 26). In addition, in music therapy this was seen when a group made music together or improvised together (24, 37). Also, in dance movement therapy this was seen in stick fight playing in which participants need to collaborate (32). These studies showed that working together in a group resulted in more pro-social behavior, more empathy, and more sensitivity to others. This could lead to more self-confidence.

### 3.3 Link to forensic models

When clustering the findings according to the Risk-Need-Responsivity model (Andrews and Bonta, 2010), the Good Lives Model (22), and the HKT-R (23), the emphasis of arts therapies interventions appears to lie primarily in the promotion of protective factors. Of the eleven clusters, eight can be linked to protective factors, six of which relate to psychological functioning (no 1, 2, 3, 6, 7, 9, 10). Three clusters can be associated with risk factors, two of which specifically address psychiatric symptoms (no 4 and 5). Only one cluster (inhibition) directly targets precursors of risk behavior such as aggression and impulsivity (no 8).

## 4 Discussion

As creative arts therapies as increasingly acknowledged in clinical and forensic contexts, and empirical studies demonstrate their effectiveness (15), the present realist review shifts the focus from whether arts therapies work to how and why they work. Eleven clusters were identified across the included studies. These clusters illustrate how active elements of the therapy, by means of underlying working mechanisms, lead to measurable outcomes. Overall, a hierarchical structure can be observed, in which the identified active elements range from those grounded in fundamental physical and bodily experiences, to those facilitating emotional expression and recognition, progressing further to elements that engage higher-order cognitive processes -such as attention regulation and executive or inhibitory functioning- and culminating in elements that foster social interaction and prosocial behavior.

More specifically, results showed that deactivation using breathing exercises, listening to relaxing music, or drumming with repetitive rhythms have been shown to lead to more relaxation, which is explained by regulating arousal. Also, creative expression and symbolization which form a central and foundational component of creative arts therapies has been shown to reduce symptoms of anxiety and depression, which is explained by better emotion regulation. Reflection and cognitive restructuring were linked to cognitive processes and strengthened cognitive control and motivation. Also, specific cognitive processes were seen in the framework of attention training improving selective and sustained attention and the framework of elements in which participants were challenged to inhibit behavior, which reduced anger outbursts. In addition, psychoeducation promoted coping skills by learning processes. Finally, two frameworks included social regulatory processes: elements in which therapists focus on attunement or group collaboration fostered empathy, prosocial behavior, and social functioning.

Our clusters confirm and extend findings from earlier work. De Witte et al. (41) proposed 19 therapeutic factor domains (active elements) across CATs, highlighting embodiment, concretization, and symbolization as unique to these modalities. Several of our clusters map directly onto these domains; for instance, deactivation corresponds to physicality of the arts and physical experience with the body, and reflection and cognitive restructuring parallel cognitive processes also identified in psychotherapy research. Additionally, discipline-specific reviews have identified active elements and proposed working mechanisms that align with our findings. For instance, Malhotra et al. (42) reviewed studies on art therapy for post-traumatic stress disorder, a prevalent condition in forensic populations. Based on a narrative synthesis, the authors identified active elements of art therapy for PTSD, including concretization and metaphor (or symbolism), active art-engagement (e.g., tactile/sensory exploration of art materials), emotion processing. Similarly, Kleinlooh et al. (43) found in their systematic review on dance-movement therapy for personality disorder that the expression and symbolization through movement and dance were key active element across included studies. It was proposed that this could help to express emotions, organize thoughts, and (re)structure personal narratives. In a review on client experiences in drama therapy, it was found that clients emphasized the importance of exploring inner experiences, accessing and releasing emotions, and enabling a sense of belonging (44). Comparable active elements have also been identified in the development of interventions. For example, De Witte et al. (45, 46) described the use of musical tempo and dynamics to (de)activation as part of a music therapy micro intervention aimed at stress reduction. While earlier studies (e.g., 41; 47, 48) described broad therapeutic domains, our findings specify observable active elements and systematically link them to mechanisms and effects. By explicating these context–mechanism–outcome configurations, the findings move beyond descriptive categories toward explanatory insights into how and why interventions work. This concreteness allows for operationalization of interventions in forensic clinical practice and for designing mechanism-focused outcome studies.

A working mechanism essentially originates in the activity of brain networks (49). Brain networks represent structural and functional regions in the brain that operate in a coordinated manner to support a range of information-processing functions (e.g. 50). Effects of creative arts therapies may be attributed to improved coordination among brain networks or the restoration of a previously dysfunctional brain network. In cases of brain network dysfunction, the underlying cause may stem from genetic predisposition, environmental influences, or an interaction between the two. Therapeutic art interventions may engage brain networks involved in attentional control, emotion processing, and sensorimotor integration, thereby enhancing connectivity and restoring functionality in regions that were previously under- or overactive. This framework provides a conceptual bridge to understanding how targeted interventions can influence core neurobiological systems underlying arousal, attention, and emotion regulation. For example, the primary brain network regulating arousal is located in the brainstem and is known as the reticular activating system (RAS). Arousal regulation has been identified as a key mechanism through which musical tempo influences physiological responses regulated by RAS, with several reviews demonstrating that variations in musical tempo modulate heart rate, heart rate variability, and blood pressure (51). Also, neurobiological evidence provides support for the plausibility of emotion regulation as a working mechanism. For example, 52 identified recurring reports of medial prefrontal cortex–amygdala involvement during creative arts engagement, consistent with broader literature implicating these circuits as active in emotion regulation. While the available evidence remains limited, these observations are in line with our proposed clusters, linking (de)activation to arousal regulation and also creative expression and symbolization to emotion regulation.

Aligning these results with forensic models, these findings show that arts therapies contribute both to the reduction of psychiatric symptoms and, more prominently, to strengthen protective factors such as social interaction, empathy, trust, coping, and self-confidence. The emphasis on protective rather than risk-reducing pathways suggests that the unique value of arts therapies in forensic settings lies in building resilience and adaptive capacities that can support desistance and long-term rehabilitation. According to the Risk-Need-Responsivity (RNR) model (Andrews and Bonta, 2010), interventions should address criminogenic needs such as impulsivity, antisocial cognition, and negative emotionality. Our clusters on inhibition, reflection, cognitive restructuring, and deactivation align directly with these domains. The emphasis on protective factors resonates with the Good Lives Model, which prioritizes the development of personal strengths and capacities to support desistance. By facilitating processes such as emotional awareness, impulse regulation, and social attunement, arts therapies provide tools that can enhance treatment motivation and engagement in a population often characterized by low insight and limited verbal communication.

### 4.1 Methodological considerations and limitations

The review synthesized evidence from a heterogeneous body of studies, including RCTs, CCTs, and pre–post designs. While this provides a broad overview, it also introduces limitations regarding comparability and generalizability. Many included studies had small sample sizes, varying intervention formats, and different outcome measures, which complicates direct comparison. Furthermore, the distribution of modalities was uneven: music therapy and art therapy were more frequently studied than dance movement therapy or drama therapy, leading to more descriptions of active elements and mechanisms for some disciplines, while others remain underrepresented. Consequently, the present review cannot offer a comprehensive overview of all possible active elements across all arts therapies disciplines.

Another important critical limitation lies in how working mechanisms and active elements were reported. Very few studies explicitly described working mechanisms, which meant that these often had to be inferred from theoretical models or clinical reasoning rather than from empirical evidence. Active elements could often be distilled from intervention descriptions, yet it remains uncertain whether these were truly the active elements, responsible for the effects, or simply core elements of the intervention. As a result, the clusters identified in this review should be interpreted as hypothesized models rather than definitive causal explanations. These models are based on a limited number of studies and should be treated as preliminary, requiring replication across a broader range of studies and further empirical testing. Future studies should therefore explicitly describe and examine active elements and its proposed working mechanisms, so that the link between intervention components and outcomes can be tested more directly and systematically.

While the adaptation of the traditional CMO framework to an ‘active element - working mechanism - outcome’ clustering allowed for a more tailored analysis aligned with our research aim, this modification may be considered a limitation, as it deviates from standard realist review methodology. Nonetheless, this approach represents an initial step toward a more precise understanding of the underlying working mechanisms within creative arts therapies. To advance this line of inquiry, future studies could integrate patient characteristics into their analyses, helping to clarify what works, for whom, and under which conditions. Employing approaches such as Bayesian network meta-analysis may offer additional insight into these complex relationships (53, 54).

This review demonstrates that creative arts therapies are effective in forensic care, with their primary strength lying in the promotion of protective factors meaning psychological and social functioning. By identifying clusters of active elements, their underlying working mechanisms and its effects, this review provides a deeper understanding of how arts therapies work and offers a theoretical basis for refining clinical practice and guiding future research. However, given the limited number of studies and the lack of explicit descriptions of active elements and working mechanisms, the models presented here should be considered tentative. Rather than providing evidence for causal pathways, they serve as a conceptual framework that illustrates how creative arts therapies may operate in forensic settings and offers directions for further replication and empirical investigation. By making these underlying processes more explicit, this review contributes to the refinement of clinical practice and provides a foundation for more targeted and systematic research into the role of creative arts therapies in forensic contexts and possibly the broader mental health care.
